# Let’s Go 3D! New Generation of Models for Evaluating Drug Response and Resistance in Prostate Cancer

**DOI:** 10.3390/ijms24065293

**Published:** 2023-03-10

**Authors:** Tina Petrić, Maja Sabol

**Affiliations:** Laboratory for Hereditary Cancer, Division of Molecular Medicine, Ruđer Bošković Institute, 10000 Zagreb, Croatia

**Keywords:** prostate cancer, therapy, resistance, 3D models, spheroids

## Abstract

Prostate cancer (PC) is the third most frequently diagnosed cancer worldwide and the second most frequent in men. Several risk factors can contribute to the development of PC, and those include age, family history, and specific genetic mutations. So far, drug testing in PC, as well as in cancer research in general, has been performed on 2D cell cultures. This is mainly because of the vast benefits these models provide, including simplicity and cost effectiveness. However, it is now known that these models are exposed to much higher stiffness; lose physiological extracellular matrix on artificial plastic surfaces; and show changes in differentiation, polarization, and cell–cell communication. This leads to the loss of crucial cellular signaling pathways and changes in cell responses to stimuli when compared to in vivo conditions. Here, we emphasize the importance of a diverse collection of 3D PC models and their benefits over 2D models in drug discovery and screening from the studies done so far, outlining their benefits and limitations. We highlight the differences between the diverse types of 3D models, with the focus on tumor–stroma interactions, cell populations, and extracellular matrix composition, and we summarize various standard and novel therapies tested on 3D models of PC for the purpose of raising awareness of the possibilities for a personalized approach in PC therapy.

## 1. Prostate Cancer

Prostate cancer (PC) is the third most frequently diagnosed cancer worldwide, and the second most frequent in men, with 1.4 million cases diagnosed in 2020. In men, it is the most diagnosed cancer in 112 countries, including North and South America, Australia, and most European and African countries. The mortality differs between the high- and low-HDI (human development index), with high-HDI countries showing higher mortality rates (37.5 per 100,000) compared to low-HDI countries (5.9 per 100,000) [1]. The 5-year survival of the early-stage disease is as high as 95%, but after the PC metastasizes, the 5-year survival drops to 30% [2]. PC most often forms metastases in the bone (84%), distant lymph nodes (10.6%), liver (10.2%), and thorax (9.1%) [3].

Risk factors for developing PC include age, family history, and specific genetic mutations. Hereditary prostate cancer (HPC) is defined with three specific parameters: (a) PC in three successive generations, (b) at least two cases of PC in the family at an age of onset <55 years, and (c) three or more first-degree relatives with PC. It is still unclear if HPC differs from the sporadic disease, but men with HPC show earlier disease onset [4]. Germline mutations may affect the development and aggressiveness of PC. A recent systematic review identified germline mutations in genes involved in homologous recombination (*BRCA1/2*, *ATM*, *CHEK2*, *NBN*), mismatch repair (*MLH1*, *MLH2*, *MSH6*), embryonic development and regeneration (*HOXB13*), and regulation of the cell cycle (*ATM*) [5,6], while a prospective screening program associated pathogenic variants of *MSH2* and *MSH6* with higher PCR incidence [7]. It has also been demonstrated that men of African descent show higher PC incidence and mortality than men of other ancestry, and reports show significant differences in genomes and transcriptomes between PC of African vs. European ancestry [8,9].

There are also several environmental risk factors that contribute to the disease, such as smoking and excess body weight. Smoking has been associated with an increased relative risk of PC [10]. A recent meta-analysis investigated the role of modifiable risk factors in lower-income countries and found an association of higher fat intake with increased PC risk and of higher vegetable intake and tea consumption with lower PC risk, while alcohol, smoking, red meat intake, and high body mass index showed a trend towards increased risk [11].

## 2. Diagnosis and Therapy of PC

Diagnosis of PC is usually done by combining the measurement of prostate-specific antigen (PSA) blood levels and digital rectal examination, followed by multi-parametric magnetic resonance imaging and biopsy if necessary [12]. However, PSA values may be affected by other factors, such as age, body mass index, prostate volume, and genetic predisposition [13]. It has been suggested that the Prostate Health Index (Phi), which is obtained by comparing relative concentrations of total PSA, free PSA, and [-2]proPSA, provides a better prediction of aggressive PC than total and free PSA [14]. Biopsy can stratify the disease into low- (T1/2, Gleason score ≤ 6, PSA ≤ 10), intermediate-, or high-risk disease. Intermediate-risk patients are then staged for metastases using MRI or PET-CT and bone scan, while high-risk patients are staged using CT and bone scan [12]. For low-risk PC, the recommended treatment option is active surveillance using PSA [15], but curative options, such as radical prostatectomy (RP), external beam radiotherapy (RT), and low-dose-brachytherapy, can be used [12]. Intermediate-risk patients have the same options as the low-risk, with the addition of neoadjuvant and concurrent androgen deprivation therapy (ADT), while the high-risk have the option of neoadjuvant ADT and/or docetaxel-based therapy or RP with pelvic lymphadenectomy [12]. If the disease progresses during ADT, it is considered castration-resistant prostate cancer (CRPC). Metastatic PC can be hormone-naïve or castration-resistant. In the case of hormone-naïve disease, ADT is combined with chemotherapy, and for the metastatic CRPC (mCRPC), chemotherapy can be combined with a second androgen receptor (AR) inhibitor [12]. BRCA-deficient mCRPC can be treated with PARP inhibitors [16,17], while mCRPC with alterations in the PI3K/AKT signaling pathway, mostly due to deletion or mutation of PTEN [18], can be treated with AKT inhibitor Ipataserib [16]. Patients with mCRPC with disease progression after treatment with enzalutamide or abiraterone could be treated with Olaparib, which has demonstrated longer progression-free survival [19]. Other novel treatment options include the PSMA-targeted radio ligand therapy (RLT), immunotherapy, or cell-based immunotherapy, but these approaches still have limited success and are currently being tested [16,20].

The emergence of CRPC following ADT is still the major problem in the therapy of PC. ADT is based on the inhibition of the androgen receptor (AR), which is the major driver of PC and, therefore, the most important drug target. AR drives metabolic reprogramming in PC compared to healthy prostate, and this reprogramming is even more pronounced in response to chemotherapy [21,22]. Clinical studies of second-generation antiandrogens, such as enzalutamide, abiraterone, apalutamide, and darolutamide, have demonstrated effectiveness in prolonging survival time and decreasing PSA levels [23,24,25,26].

Mechanisms leading to PC progression and ADR resistance are usually associated with amplifications, mutations, altered splicing, or epigenetic reprogramming in the *AR* gene [27,28,29]. Another potential driver is the increase in androgen biosynthesis, which can occur through the mutation in the *HSD3B1* gene [30]. Mutations in other genes may also contribute to the risk, and a panel of four genetic mutations (*MSH2*, *CDK12*, *TP53*, and *RB1*) has been proposed as a predictor of risk for early progression [31]. Complex chromosome rearrangements, or chromoplexy, which include many cancer-associated genes, have been associated with prostate cancerogenesis [32]. Therefore, many drugs are being repurposed for new application in treating CRPC [33]. New drugs and combinations of drugs designed to bypass this resistance are being tested in vitro and in vivo, such as selective AR degraders [34], AR-GR (glucocorticoid receptor) dual antagonists [35], proteolysis-targeting chimeras (PROTACs) [36], or targeted delivery of radioligands [37].

Drug testing has been traditionally performed on 2D cell cultures, where cells are grown in a monolayer attached to the surface of the dish. This model is convenient and easy to maintain, and still largely used in many laboratories worldwide. However, this model does not represent the 3D architecture of the tumor found in vivo, nor its complexity regarding the many cell types found within the tumor mass or surrounding it. The role of the tumor microenvironment has been identified as critical in facilitating tumor growth, especially the cancer-associated fibroblast population [38]. Therefore, the use of advanced 3D models is coming into focus as more biologically relevant for the purpose of identification of novel factors contributing to PC tumorigenesis and resistance, but also for drug testing.

## 3. Three-Dimensional Prostate Cancer Models in Drug Discovery

### 3.1. Spheroids vs. Organoids vs. Tumoroids

Spheroid and organoid cultures are 3D cultures composed of multiple cells with distinct and overlapping purposes. They can be useful in 3D cell research, but differ in cellular sources and protocol for establishment [39]. Spheroids are spherical clusters of broad-ranging cells, usually generated from established cell lines. They are generally cultured as free-floating aggregates, with no need for a scaffolding environment to form 3D cultures. They are considered as structures of low complexity in mirroring tumor organization, but are very popular, as they emulate properties of solid tumors in several aspects [40]. Nevertheless, they are not able to self-assemble or regenerate, and thus are not as advanced as organoids. Even though cell–cell and cell–ECM interactions are present in the spheroids, when they are larger than 500 μm, they represent non-vascularized or poorly vascularized tumors [40]. They consist of proliferating cells in the outer layer, with quiescent cells in the middle, and hypoxic and necrotic cells in the inner layer, and the metabolites are distributed in a gradient through the structure. The term prostasphere stands for self-associated PC cell lines in suspension that grow as unattached spheroids, therefore, meaning that the terms prostaspheres and PC spheroids are being used interchangeably [41,42].

Organoids are complex clusters of organ-specific cells, either stem cells or progenitor cells. Together with a given scaffolding extracellular matrix, or collagen, they can self-assemble. Histologically and genetically, they resemble the original tumor from which they were derived, both in structure and function [39,43]. They can be cultured from a very small amount of tissue and are easy for genetic manipulations [44,45]. Organoids can be maintained in long-term culture and can be cryopreserved [39].

The difference between organoids and tumoroids is that organoids lack the full complement of cells and factors found in a patient’s tumor, while tumoroids retain the full architecture of the tumor microenvironment (TME) and extracellular matrix (ECM). The cells included in the tumoroid include multiple support cells, such as cancer-associated fibroblasts (CAFs), endothelial cells, and pericytes; immune cells, such as lymphocytes, neutrophils, dendritic cells (DCs), and monocytes; and less-prevalent cells, such as myeloid-derived suppressor cells (MDSCs), mesenchymal stromal cells (MSCs), and platelets [46]. This is due to the fact that tumoroids are grown directly from fresh patient tumor tissue. Maintaining the TME and ECM is of great importance, especially in drug testing studies, because it provides the true response to conventional chemotherapeutic and targeted therapies [47].

Spheroids, in general, and prostate spheroids, as well, show upregulation of stemness markers, such as CD44, GLI1, ABCG2, and BMI1 [48]. Generation of spheroids can be used as a step in the process of enrichment of cancer stem cells (CSCs) from a cell population. Spheroids are formed, then disassociated and sorted for specific markers of stemness (e.g., CD44, CD133), thus obtaining a CSC-rich population of cells [49], and this can be applied to primary PC cultures and clinical samples, as well [50,51].

### 3.2. Methods for Growing 3D PC Models

#### 3.2.1. Suspension Cell Cultures

**Figure 1:**
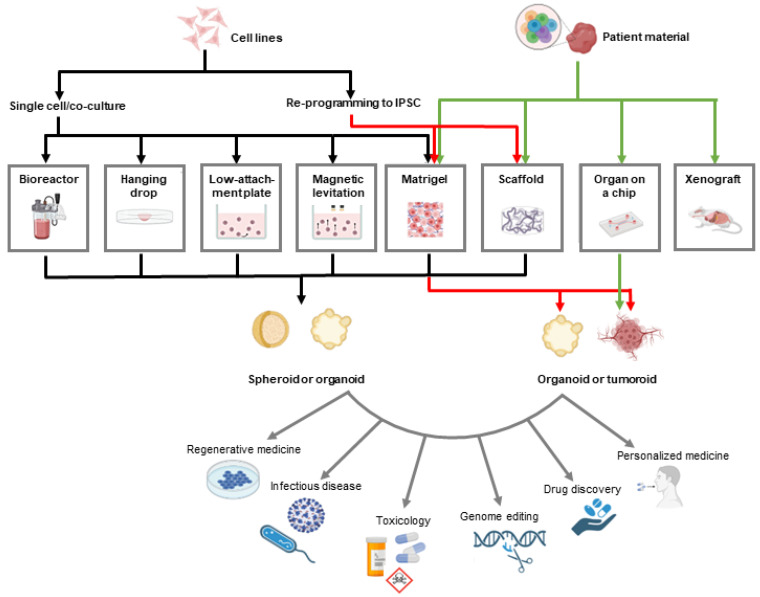
Outline of different methods for generating 3D models. If the starting material is cell lines, they can be grown as single cultures or co-cultures to generate spheroids or reprogrammed to IPSC and then differentiated in vitro using matrices and scaffolds to generate organoids. If patient material is used, it is usually dissociated into single cells before plating onto scaffolds, matrices, or chips to generate organoids or tumoroids. Patient material can also be implanted into animal models as xenografts. The prepared 3D cultures can then be used for a variety of applications, as presented in this schematic. Created in Biorender.com.

There are several methods to develop 3D models in PC (Figure 1). The first and most popular is the self-assembly of PC cells in non-adherent culture conditions, which limits attachment of the cells on surfaces, for instance, by using agarose coating or non-adherent plastic dishes. This method is simple, low-cost, and offers a consistent yield, and it is suitable for multicellular spheroids [52]. However, some limitations lie in the difficulty controlling the spheroid size and the lack of extracellular matrix (ECM) surrogates. The uniformity of the spheroids can be achieved by generating microwells on the surface of the agarose layer, which enables generation of spheroids of a specific size [53], or by seeding a defined number of cells in each well of the round-bottomed specialized plates. In addition, this model represents problems with drug testing since it is not suitable for migration/invasion, and no cell viability assay has been developed so far [54,55,56,57,58,59,60,61]. Therefore, this model is most often used for the spheroid formation assay, where the number and size of spheroids is compared between non-treated and treated cells. A large-scale approach for this model includes the use of bioreactors with the rotating wall vessels, where a large number of suspension spheroids can be cultivated [62]. Spheroids can also be generated with microgravity, and PC cells exposed to microgravity separate into two populations, the adherent cells and the spheroids in the suspension above the adherent cells [63,64].

#### 3.2.2. Hanging Drop

The hanging drop method is the second approach, very similar in its characteristics to the suspension culture. Cancer cells are seeded and incubated in hanging drops until they form rounded structures characterized by stable cell–cell contacts [65]. The drops can be formed by hanging the cell suspension from the lid of a petri dish, or by using specialized plates. Some advantages of this approach are the small starting numbers of cells and media volumes, uniform spheroid size that can be adjusted as necessary by modifying the number of cells during seeding, and possibility for the co-culture of different cell types [66]. However, drawbacks of this method are the difficult medium exchange, which limits drug addition; the lack of extracellular matrix surrogates; and no possibility for migration/invasion or cell viability assays [66].

#### 3.2.3. Organ-on-a-Chip Technology

An organ-on-a-chip is a more complex approach that enables PC cells to recreate in vitro the architecture of in vivo tumor mass, which is based on microfluidic devices [67]. Microchip manufacturing methods used for this approach contain continuously perfused chambers inhabited by living cells arranged to simulate tissue- and organ-level architecture [67]. This device produces levels of tissue and organ functionality not possible with conventional 2D or 3D culture systems. Within this system, it is possible to incorporate various cell types equally distributed within the chip, and they can be kept still during media exchange. Organ-on-a-chip enables high-resolution, real-time imaging and in vitro analysis of biochemical, genetic, and metabolic activities of living cells in a functional tissue and organ context, as well as the study of tissue development, organ physiology, and disease etiology [67]. Spheroids generated in this manner are uniformly sized, and their formation is fast, with constant perfusion and uniform distribution of oxygen and nutrients. However, a drawback of this approach would be the necessity of specialized equipment and expensiveness [68].

#### 3.2.4. Gel-Embedding

Some additional models called gel-embedding models include extracellular matrix-like gels, such as highly hydrophilic polymers with a soft tissue-like stiffness designed to mimic the extracellular protein network. Such gels include Matrigel, alginate, and collagen. Advantages of this approach are the formation of contacts between cells and the artificial extracellular matrix, as well as the possibility to perform migration/invasion assays. This model is also known as the liquid-overlay method, as the cells are first embedded in the matrix, and then the pellet is overlaid with the culture media. However, some disadvantages are the undefined composition of natural gels and the structural modification over time [69,70,71,72,73,74], as well as their impact on the lower penetration of the drugs to the destination cells, leading to lower drug efficacy [75].

#### 3.2.5. Prefabricated Scaffolds

Prefabricated scaffolds can be considered a replacement for the ECM. They can be made of natural (i.e., collagen) or synthetic (i.e., polycaprolactone) polymers, and they create a porous environment for the physical support and growth of spheroids. Even though they ensure high tissue mimicry and maximum resemblance to the in vivo conditions, with the possibility of the use of a wide variety of materials and properties, they are much more complicated and expensive than those for gel production, and there is a risk of possible variability between scaffolds [76,77,78]. Furthermore, as in the case of Matrigel, scaffold composition may lead to spatially divergent treatment effects [75].

#### 3.2.6. Patient-Derived Explants

The most advanced model is the ex vivo 3D culture of freshly excised PC specimens, called patient-derived explants (PDEs). This approach developed as an alternative to the use of immortalized PC cell lines to test the efficacy of new drugs in vitro or in vivo. This is basically the cultivation of tissue pieces or slices on sponge scaffolds. The advantages of this approach, besides low cost, are high tissue mimicry and direct assessment of patients’ therapeutic responses on an individual sample, which is useful for development of personalized medicine strategies. Of course, there is a limitation in the sense of reliance on fresh tissue and specialized equipment, and expertise, as well [79,80,81,82,83].

### 3.3. Drug Discovery and Screening

Drug discovery and screening is, in 3D cultures, most often limited to monitoring the spheroid-forming capabilities of cells exposed to treatment compared to solvent control. The size, shape, and number of spheroids are measured and compared, providing information about the effectiveness of the used substance in this setting. Many groups use proliferation and viability assays to evaluate the fitness of their spheroids, although IC50 can be an imperfect index for evaluation of spheroid viability, and the response to the same drug can differ based on the type of spheroid model (floating vs. matrix-embedded) and on the size/uniformity of spheroids [84]. Some studies go beyond this and perform sectioning and immunofluorescent staining of specific targets of interest, measure gene/protein expression in the 3D models, and even measure some metabolic parameters of performed RNA sequencing. An overview of the recent studies on PC cell lines involving spheroid/organoid/tumoroid models using different therapeutics is presented in Table 1.

Cell-based assays are still the main tool for testing the efficacy of a new compound in drug discovery. While comparing 2D and 3D cell models, it has been shown that there are some remarkable differences between the two [85]. Cellular responses to drug treatments in 3D models are for sure more similar to in vivo responses when compared to 2D models. For instance, 3D cell models are more resistant to the anticancer treatment than 2D models, which has been demonstrated for several different cancers and combinations of drugs used in these types of cancer [86,87,88,89,90]. It has been shown that drugs were often highly active in 2D models, while less active and gradually lost their activity in 3D spheroids/organoids. This would imply that certain geno- and phenotypical changes induced by 3D spheroids/organoids formation are responsible for increased drug resistance due to the signals from dynamic cellular interactions between neighboring cells and ECM input into the cellular decision-making process [87,91]. Increased drug resistance is probably due to the limited diffusion through the spheroid/organoid, which leads to the drug concentration gradient across a single spheroid/organoid and hypoxia, which has been shown to lead to the activation of genes involved in cell survival and drug sensitivity [75]. Moreover, stromal cells also have been shown to be involved in the drug resistance, and this chemoresistance is observed in vivo, as well [87,92]. Several clinical trials failed upon reaching advanced stages of drug testing due to the fact that sometimes resistance mechanisms are not active in 2D cell culture, but are seen in 3D cell culture models, as well as in vivo in a xenograft model [85].

Differences in physical and physiological properties of 2D and 3D models affect their response to the drug treatment. For instance, 2D cells are stretched out on a flat substrate, while 3D cells on a natural or synthetic scaffold material maintain normal morphology and multiple contacts with the surrounding cells, and these differences in the morphological spread contribute to the differences in the drug response between the two. Moreover, the difference in the expression and the spatial organization of surface receptors in these two models also affects the response to drugs since the levels of receptors and the binding efficiency of a drug to these receptors is different due to the difference in the structure, localization, and spatial organization of these receptors on cell surfaces [93,94]. In addition, there is a difference in cancer gene expression levels because in 2D cell culture, some genes are differently expressed, which can lead to the different response and, thus, affect the effectiveness of a drug [93,95]. Moreover, we need to take into consideration that cells in 2D and 3D are also in different stages of the cell cycle, meaning that 2D cells are mainly proliferative, while 3D cells are usually a mixture of cells in proliferation, quiescence, and even apoptosis/necrosis, having proliferating cells on the outer region and quiescent cells in the middle region due to the lack of nutrients and gas exchange, while the center of the 3D structure often contains dead cells [96]. Further, active cell proliferation is sometimes required for drugs to be effective, so only the outer layer of the 3D structure will be affected, while the quiescent cells of the middle layer may respond poorly [97].

Drug accessibility to cells and local pH is also important for the difference in drug response. In the 2D monolayer, drugs diffuse to cells equally [98]. In the 3D model, diffusion of the drugs is dependent on the distance of the cells from the surface and the local pH [99]. Both hypoxia and lower pH contribute to the drug resistance, as there is no efficient transport system to remove waste from the central region of the spheroid. Additionally, lower pH can reduce the efficiency of the uptake of the drug, resulting in increased resistance [100]. The use of patient-derived primary tumor cells for the generation of 3D models proved to be promising in evaluating cellular responses to antiproliferative cytotoxic and targeted agents, as well as in assessing the chemosensitivity and signaling pathway activity in cancer cells. This was demonstrated in some of the most common cancers, including lung, breast, and prostate cancers [98]. These findings paved the way for patient-derived 3D models in the development of personalized medicine, as the same model can be examined in vitro and in vivo for the analysis of various signaling pathways and evaluation of chemosensitivity [101,102,103].

**Table 1:** Recent examples of the use of 3D in vitro models for testing of various compounds and therapies.

Type of Treatment	Name of the Compound/Treatment	Type of 3D Model Used	Reference
chemotherapeutic	docetaxel	spheroids in U-bottom plates and Matrigel-embedded	[104]
chemotherapeutic	docetaxel on gold nanoparticles	spheroids in low-attachment plates	[105]
chemotherapeutic	bortezomib	spheroids in agarose-coated plates	[106]
chemotherapeutic	docetaxel on microparticles	spheroids in low-attachment plates	[107]
natural compound	Brachydin C	spheroids in agarose-coated dishes	[108]
natural compound	Brachydin A	spheroids in agarose-coated dishes	[109]
natural compound	green tea extract	spheroids in hanging drop	[110]
natural compound	perillilaldehyde	spheroids in poly-HEMA-coated plates	[111]
natural compound	pristimerin	spheroids in poly-HEMA-coated plates	[112]
natural compound	curcumin	spheroids in low-attachment plates	[113]
natural compound	gallic acid	spheroids in hanging drops	[114]
natural compound	procyanidin B2 3,3″-di-O-gallate	spheroids in low-attachment plates	[115]
natural compound	rosmarinic acid	spheroids in hanging drops	[116]
statin	simvastatin	spheroids in hanging drop (plates)	[117]
statin	rosuvastatin	spheroids in agarose-coated plates (liquid overlay)	[118]
ADT	darolutamide	spheroids in low-attachment plates	[119]
radionuclide	radium-233	spheroids in low-attachment plates	[120]
radionuclide	^225^Ac on liposomes/antibody	spheroids in low-attachment plates	[121]
radionuclide	^64^CuCl_2_	spheroids in low-attachment plates	[122]
hormone	17β-estradiol or testosterone	spheroids in agarose-coated wells (1 spheroid/well)	[123]
antibody	TNB-585 (anti-PSMA antibody)	spheroids in low-attachment round-bottom plates	[124]
antibody-drug conjugate	antibody-drug conjugate U3-1402	patient-derived xenograft organoids	[125]
antibody-drug conjugate	antibody-drug conjugates VH1-HLE-AF680	spheroids in methocellulose + Matrigel hanging drop plates	[126]
ligand-radionuclide conjugate	PSMA-targeting ligand labeled with ^212^Pb	spheroids in agarose-coated plates	[127]
immunotoxin	anti-PSMA immunotoxin hD7-1(VL-VH)-PE40	spheroids in agarose-coated plates	[128]
oncolytic virus	PIV5 oncolytic virus	spheroids in low-attachment plates	[129]
ultrasound	focused ultrasound	spheroids in low-attachment plates	[130]
microgravity	microgravity	spheroids in microgravity or agarose-coated dishes	[63]
CHK1 inhibitor	MU380	spheroids in low-attachment plates	[131]
DNMT inhibitor	CM-272	spheroids in U-bottom plates	[132]
kinase inhibitor	ponatinib, sunitinib, sorafenib	organoids	[133]
kinase inhibitor	Dovitinib, BGJ398, or PD166866	spheroids in agarose-coated plates	[134]
HDAC inhibitor	Jazz90, Jazz167	spheroids in Matrigel	[135]
mPGES-1 inhibitor	KH176m	spheroids in Matrigel, low-attachment plates	[136]
TRPM8 antagonist	TRPM8 antagonist	spheroids in ECM	[137]
NUAK antagonist	WZ4003 and HTH-02-006	spheroids in low-attachment plates	[138]
PKC agonist	HMI-1a3	spheroids in agarose-coated U-bottom plates	[139]
Cyclodextrin nanosponge	GSH-NSs	spheroids in hanging drops	[140]
cytotoxic metal	Ir(III)–Cu(II) Compounds on liposomes	spheroids in hanging drops	[141]
cytotoxic metal	IrIII complex conjugated to coumarin	spheroids in low-attachment plates	[142]
glycoprotein	fetuin-A	spheroids in low-attachment plates	[143]
peptide	GV1001 peptide	spheroids in low-attachment plates	[144]
small molecule	ATPγS and ATP	spheroids in spheroid culture plates	[145]

## 4. Standard and Novel Therapies Used in 3D Models of PC

Results obtained so far from different 3D assays and approaches used in PC early-stage drug discovery encompass radiotherapy, hormone therapy, chemotherapy, targeted therapies, and novel and experimental therapies [146]. Many combined therapies, which include different approaches, are also being tested, and the most recent studies are summarized in Table 2.

### 4.1. Radiotherapy

Radiotherapy is recommended for localized or locally advanced PC [147]. The report of Camus et al. on the viability of 3D multicellular PC spheroids after treatment with Surface Enhanced Raman Spectroscopy (SERS) showed that this novel method for measuring intracellular redox potential and pH in 3D live cultures can actually represent a potential new platform for in vitro preclinical characterization of tumor models [148]. Radiation can be applied on 3D cultures in combination with potential sensitizers, for example, AMPK activator AICAR [149], or cytotoxic metals [150]. Apart from the classic irradiation, there is some interest in targeted delivery of radionuclides to the PC by using different targeting molecules, and this approach is also being tested in vitro on 3D cultures. Different radionuclides and isotopes, such as ^233^Ra, ^225^Ac, ^212^Pb, or ^64^CuCl_2_, have been tested on spheroid models, either directly or using different carriers, and showed a good effect on spheroid models [120,121,122,127]. ^233^Ra pre-treatment of HAp surface has shown a drastic effect on the survival of PC cells and spheroid outgrowth [120]. Spheroid cultures can be used to assess the effectiveness of delivery and to test different carriers, as was demonstrated by Salerno et al.: ^225^Ac α-particles were delivered by either tumor-responsive liposomes or antibodies, and they have shown that small spheroids (80–100 μm) were more effectively inhibited by the radiolabeled antibodies, large-size spheroids (300 μm) were more responsive to liposome carriers, while the combination of both carriers was the most effective for intermediate-size spheroids (200 μm) [121].

### 4.2. Hormone Therapy

Treatments that reduce androgen production by the testicles are the most commonly used hormone therapies for PC. ADT can be performed surgically or chemically. The surgical option is orchiectomy, a procedure to remove one or both testicles, which can reduce the level of testosterone in the blood by 90% to 95% [151]. The remaining 5% is produced by the adrenal gland, so luteinizing hormone-releasing hormone (LHRH), also known as gonadotropin-releasing hormone (GnRH), agonists are used (goserelin, triptorelin, histrelin). They produce an initial surge in luteinizing hormone (LH) and testosterone levels, but constant exposure to LHRH desensitizes the pituitary cells and, therefore, suppresses testosterone levels [152]. LHRH/GnRH antagonists, such as degarelix, abarelix, and relugolix, can also be used, as they inhibit downstream LH signaling and achieve chemical castration within 2–3 days [152]. Antiandrogen therapies, treatments that block the action of androgens in the body, are not considered typical ADT and are often used concurrently with ADT or when ADT stops working. Such treatments include androgen receptor blockers (also called androgen receptor antagonists), which compete with androgens for binding to the androgen receptor, or androgen synthesis blockers, which prevent the production of androgens. Some examples of the androgen receptor blockers include the first-generation drugs flutamide, bicalutamide, and nilutamide, and the second-generation drugs enzalutamide, apalutamide, and darolutamide [153]. Some of the approved androgen synthesis inhibitors are abiraterone, ketoconazole, and aminoglutethimide. Abiraterone is also approved in combination with prednisone to treat metastatic PC, both castration-sensitive and castration-resistant [154].

Advanced CRPC represents a major clinical problem since the gold standard, AR targeting, is not as effective in the long run as previously thought [155,156]. Therefore, a co-culture 3D model of PC and CAF has been developed, and it was demonstrated that the stromal cells reduced the sensitivity of PC cells to androgens and other drugs without altering AR levels. This has demonstrated that this kind of PC and CAF combined 3D model is necessary to understand how CAF can influence the drug response of PC cells to current therapies. Therefore, this type of 3D co-culture can be a valuable in vitro drug-testing tool [157].

### 4.3. Chemotherapy

Chemotherapy is used in advanced PC, CRPC, or mCRPC. Several standard drugs are used as chemotherapeutic agents for these advanced stages of PC. Standard chemotherapy begins with docetaxel combined with prednisone [158]. However, there are some recent advances in this kind of treatment. Addition of hormonal therapy to docetaxel in those with newly diagnosed PC, or the use of cabazitaxel to treat mCRPC, showed significant reduction in tumor growth and spread. Some new approaches, such as using drug-encapsulating polymersomes, which contain docetaxel and present folic acid on the surface, or micellar delivery systems for paclitaxel, have demonstrated higher cytotoxicity than using the drug in free form [159,160]. The advantage of such delivery systems is their ability to penetrate into the center of the spheroid/tumor mass, bypassing the issue of reduced drug permeability and uptake [146]. Tumor cells can be sensitized to chemotherapy by using various compounds, e.g., MU380 can sensitize docetaxel-resistant PC to gemcitabine; MF-15 can re-sensitize enzalutamide-resistant cells to anzalutamide; and paxilline can reverse the resistance to docetaxel, palitaxel, doxorubicin, and cisplatin. [117,131,161]. Some compounds can also act synergistically, such as resveratrol with docetaxel [162] or JQ1 with docetaxel [104].

### 4.4. Targeted Therapies

Targeted therapies are designed to stop only the growth of the cells with a specific mutation, in this way sparing the healthy cells from damage. This kind of treatment is different from chemotherapy, which damages healthy cells, along with the cancer cells [163]. There are several agents used for this kind of treatment. ANTI-ANGIOGENIC AGENTS, such as Aflibercept, Bevacizumab, and Thalidomide/Lenalidomide, target angiogenesis, which is an important process for the growth, progression, and metastasis of solid tumors [164,165,166]. TYROSINE KINASE INHIBITORS (TKIs) inhibit tyrosine kinases alone or in combination with other targets, such as the angiogenesis factors mentioned before. Tyrosine kinases are mediators of intracellular signaling pathways that control cell growth, migration, and invasion. Some examples in this group include Dasatinib, Cabozantinib, and Sunitinib [133,134,167]. ENDOTHELIN RECEPTOR ANTAGONISTS are Atrasentan and Zibotentan. Endothelins are small proteins implicated in tumor growth and metastasis [168]. CLUSTERIN INHIBITORS (Custirsen) inhibit clusterin, a cytoprotective chaperone whose transcription is promoted by the androgen receptor and heat shock Factor-1, a key mediator of carcinogenesis [169]. Other agents worth mentioning are: B-cell Lymphoma 2 (Bcl-2) inhibitors, Insulin-Like Growth Factor (IGF) inhibitors, inhibitors of phosphatidylinositol 3-kinase (PI3K)/AKT/mammalian target of rapamycin (mTOR) pathway, and immunotherapeutic agents. The mammalian target of rapamycin (mTOR) is a serine/threonine kinase that regulates cell growth and cell cycle progression and integrates signals from growth factors and is aberrantly activated and frequently mutated in PC [170,171,172]. HDAC inhibitors have also demonstrated potential in the 3D model [135]. Targeted therapies are being tested with currently known chemotherapeutics or natural compounds in an effort to either increase the sensitivity of PC to known drugs or to re-sensitize resistant tumors (recent publications are summarized in Table 2). Some molecular targets have been tested in a 3D in vitro setting, and have been shown to affect the sensitivity of PC spheroids to treatment, for example, CD133 suppression increases sensitivity to paclitaxel [173], CDH1 loss sensitizes PC to DNA-damaging agents [174], and midkine downregulation sensitizes cells to quercetin [175]. In summary, targeted therapies demonstrate high tumor cell specificity and efficacy, while providing acceptable toxicity and side effects [164]. However, targeted therapy of mCRPC is still not showing satisfactory outcomes, and there was no difference in survival when docetaxel or prednisone were used, with or without targeted therapy [164].

### 4.5. Novel and Experimental Therapies

Many natural compounds are being tested for their effect on PC, and 3D spheroid models are used as either simple spheroid formation assays to assess the ability of the cells to form spheroids after treatment, or more complex studies that include measuring viability, apoptosis, metabolic parameters, and gene and protein expression. Curcumin is one of the most investigated natural compounds in this context, and it has demonstrated anti-tumor effects on many tumor types, including prostate [113,176,177]. However, the search for new natural inhibitors continues, as many are being tested on PC spheroids, such as flavokawain A [178], brachydin A [109], green tea extract [110], rosmarinic acid [116], or grape seed extract [115].

3D cultures are being used to develop novel carrier systems with the possibility to penetrate deeper into the structure of the tumor and deliver specific compounds, such as radionuclides, miRNA, and drugs. Several carriers have been tested that have shown increased permeability, even in 3D cultures [107,121,126,141,142,179,180].

MicroRNA (miRNA) molecules can be used to target specific genes of interest; however, their application in vivo remains controversial, as precise delivery to target cells is required to avoid off-target effects. The combining of specific miRNA with standard therapy is showing great potential in vitro, including on 3D cultures. siCD133 combined with paclitaxel shows a synergistic effect in vitro and inhibits spheroid formation [173]. siMRP1 combined with doxorubicin and loaded onto silicon nanoparticles shows increased retention and selectivity to the tumor in 2D and 3D conditions [181]. siEphA2 combined with a small-molecule HDAC inhibitor loaded into lipid nanoparticles induces cytotoxicity in 3D spheroids comparable to that observed in the 2D monolayer [167]. siMK in combination with quercetin results in reduced spheroid size compared to either treatment alone [175].

**Table 2:** Recent examples of the use of 3D in vitro models for testing of combinations of therapies.

Type of Treatment	Name of the Compound/Treatment	Type of 3D Model Used	Reference
chemotherapeutic + natural compound	lactic acid, arctigenin, docetaxel	spheroids in low-attachment plates	[182]
chemotherapeutic + natural compound	curcumin, cisplatin, paclitaxel, docetaxel	spheroids in Matrigel	[177]
chemotherapeutic + BET inhibitor	JQ1, docetaxel	spheroids in Matrigel	[104]
chemotherapeutic + PARP inhibitor	olaparib and carboplatin	PDX-derived organoids for drug sensitivity testing	[183]
chemotherapeutic + radiotherapy	carboplatin and radiotherapy	organoids	[174]
chemotherapeutic + siRNA	siMRP1 + doxorubicin	spheroids in low-attachment plates	[181]
chemotherapeutic + siRNA	siCD133 + paclitaxel	spheroids in Matrigel	[173]
chemotherapeutic + TRAIL inhibitor	taxanes + TRAIL	spheroids in low-attachment plates, monocultures or mixed with fibroblasts	[184]
chemotherapeutic + uricosuric	probenecid, doxorubicin, cisplatin	spheroids in low-attachment plates	[185]
chemotherapeutic + antioxidant	resveratrol + docetaxel	spheroids in low-attachment plates	[162]
chemotherapeutic + hypoxia-activated prodrug	docetaxel, TH-302	spheroids in low-attachment plates	[100]
chemotherapeutic + antibiotic	ciprofloxacin, doxorubicin		[186]
chemotherapeutic + NAMPT inhibitor	FK866 + doxorubicin	spheroids in bioreactor	[187]
chemotherapeutic + ion channel inhibitor	Paxilline + docetaxel, paclitaxel, doxorubicin, and cisplatin	spheroids in low-attachment plates	[161]
ADT + anti-inflammatory drug + AKR1C inhibitor	MF-15, indomethacin, enzalutamide	spheroids in low-attachment plates	[117]
ADT + cytokine	IL-23, enzolutamide, darolutamide	spheroids in low-attachment plates	[188]
ADT + small peptide	small peptide Rh-2025u, enzalutamide	spheroids in Matrigel	[189]
ADT + small peptide	Enzalutamide or Bicalutamide, recombinant NRG1 peptide	organoids, xenograft	[190]
natural compound + MEK inhibitor	curcumin, PD98059	spheroids in low-attachment plates	[176]
natural compound + NEDD8 inhibitor	flavokawain A, MLN4924	spheroids in low-attachment plates	[178]
acyl-CoA synthetase inhibitor + contrasting agent	5-aminolevulinic acid, triacsin C	spheroids in low-attachment plates	[191]
cytotoxic metal + radiation	[Cu(TPZ)2]-liposomes and gamma-radiation	spheroids in agarose-coated plates	[150]
hyperthermia + radiation	hyperthermia + electron radiation	spheroids in low-attachment plates	[192]
NDRG1 inhibitor + iron chelator	thiosemicarbazones, Dp44mT, DpC	spheroids in collagen hydrogel (liquid overlay)	[193]
OGT inhibitor + CDK inhibitor	OSMI-2 + AT7519	spheroids in Matrigel	[194]
kinase inhibitor + siRNA	siEphA2, JIB-04 in lipid nanoparticles	spheroids in poly-HEMA-coated plates	[167]
AMPK activator + radiation	AICAR + radiation	spheroids in agar-coated plates (liquid overlay)	[149]
statin + anticonvulsant	valproic acid, simvastatin	spheroids in low-attachment plates, multiple generations	[195]
antioxidant + siRNA	siMK + quercetin	spheroids in agarose-coated plates (liquid overlay)	[175]

Finally, an important feature to investigate during PC development and metastasis is the spread of PC to the bone [196]. PC most often metastasizes to the bone, and this is the primary cause of PC-related morbidity and mortality [197]. Certain translational 3D models have been developed to mimic the complex structure of the cancer metastasis, and some valuable recent examples include breast and PC [98]. A biomimetic bone microenvironment was designed to mimic the mesenchymal-to-epithelial transition (MET) of PC using highly metastatic and non-metastatic PC cell lines [169,170,198]. Bioactive factors from osteogenic induction of human mesenchymal stem cells (MSCs) were added to the porous 3D scaffold of different compositions (e.g., PLGA, nano-hydroxyapatite (nHA)/PLGA [168], and nanohydroxyapatite/collagen mixed scaffolds [171]). Such structures mimicked the interaction between the PC and bone microenvironments and allowed for the study of novel therapeutic approaches. In addition, genetically engineered mouse models (GEMMs) that mimic PC bone metastasis can be used as an efficient model for studying the advanced stages of PC in vivo [199,200]. Until now, the improvements in the treatment of PC have mainly been made in early-stage localized disease. However, the distinction between indolent and aggressive tumors and the lack of efficient therapies of advanced PC still represent a major problem in PC management. GEMM can offer the possibility to generate new models that accurately reflect human disease and to implement this knowledge in drug discovery and screening [201]. So far, this model has been used mainly for the investigation of PC tumor initiation and progression, with little or no focus on PC metastasis to the bone. Nevertheless, one of the studies established a prostate-derived tumor line that showed frequent metastasis to the bone and growth in an immunocompetent host [202]. In this way, a useful model was generated to study the mechanisms of bone metastasis, as well as the tumor immune response. Sadly, the publications on this topic are still sparse, and there is room for expansion in this area.

## 5. Conclusions and Future Directions

Cancer studies are mainly relying on in vitro models, and continuous improvement of these models is crucial for the further development of cancer research. Therefore, an upgrade to the 3D model architecture is the future of PC research in order to better understand the complex mechanisms influencing PC development and progression. This is essential for more comprehensive drug discovery and screening. Novel therapies are much needed, especially for CRPC and mCRPC. Research into new 3D models, which are able to closely reflect the tumor microenvironment, have shown impressive progress in the last decade, and many options have been developed for 3D model establishment and maintenance. It is of great importance to mimic the natural growth of cancer more closely, and in this sense, it is necessary to engineer separate cancer masses and biomimetic stromal compartments containing appropriate cell populations (e.g., fibroblasts, endothelial cells, immune cells, and other ECM components). Heterotypic spheroids, organoids, and tumoroids are models of increasing complexity that take into account the contribution of ECM and supporting cell populations. Further attention should be given to the primary cancer cell lines and PDX models because this could lead to the development of personalized drug-screening platforms. The future of 3D PC research lies not only in the investigation of tumor growth, but also in studying invasion, migration, cancer stem cells plasticity, and cancer cell dormancy, which can model the interaction between cancer and stromal cells more accurately.

## Data Availability

The study did not report any data.
